# Supplementary figures and images for: Diversity in Notch ligand-receptor signaling interactions
Source: eLife. 2025 Jan 3;12:RP91422. doi: 10.7554/eLife.91422 (PMC11698495; doi:10.7554/eLife.91422)

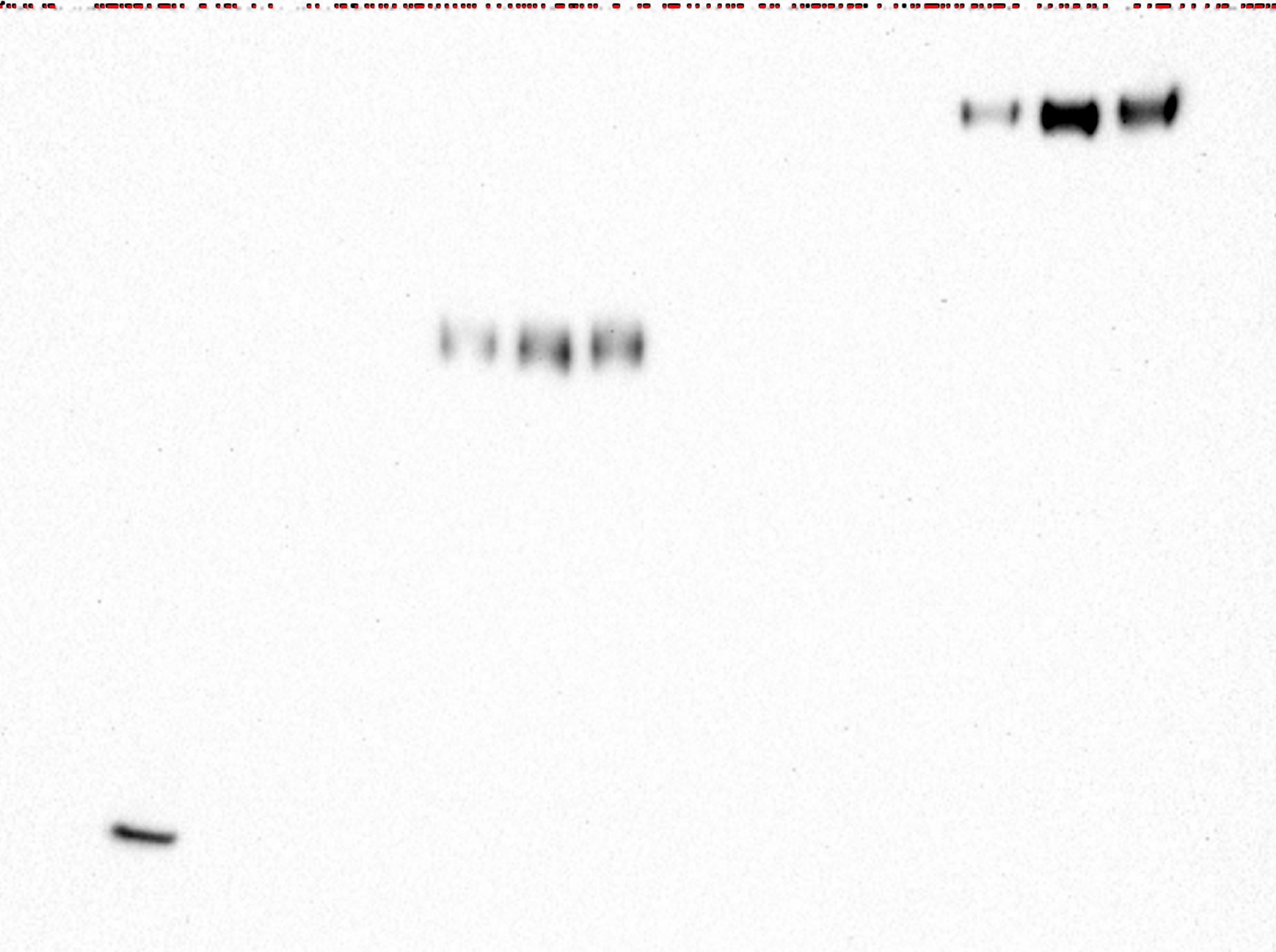

Supplement: Figure 2—figure supplement 2—source data 1. [file elife-91422-fig2-figsupp2-data1.zip › Figure2-S2B_SourceData1/Figure2-S2B_ligands_pico_120s.tif]

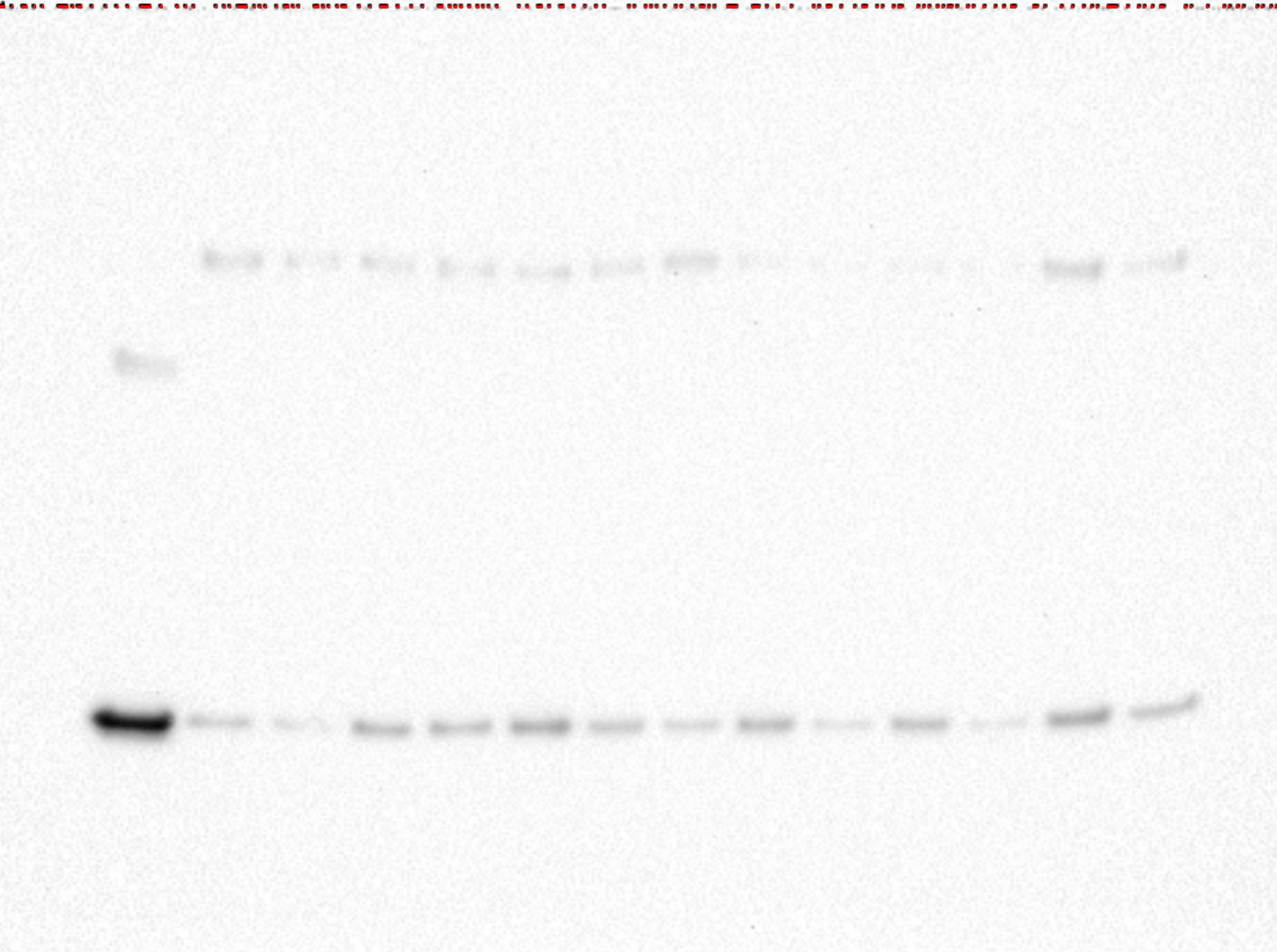

Supplement: Figure 2—figure supplement 2—source data 1. [file elife-91422-fig2-figsupp2-data1.zip › Figure2-S2B_SourceData1/Figure2-S2B_GAPDH_pico_10s.tif]

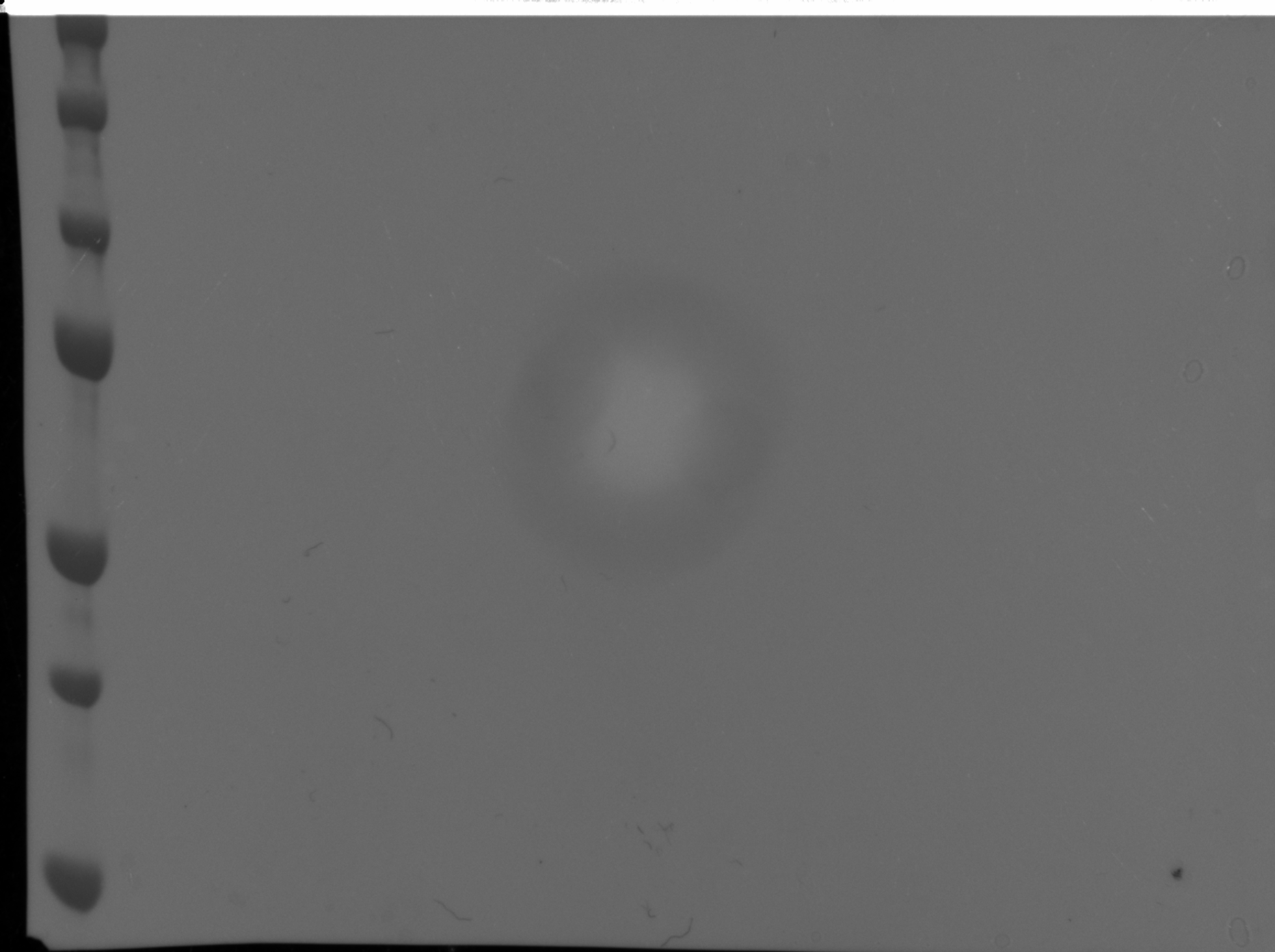

Supplement: Figure 2—figure supplement 2—source data 1. [file elife-91422-fig2-figsupp2-data1.zip › Figure2-S2B_SourceData1/Figure2-S2B_ladder_colorimetric.tif]

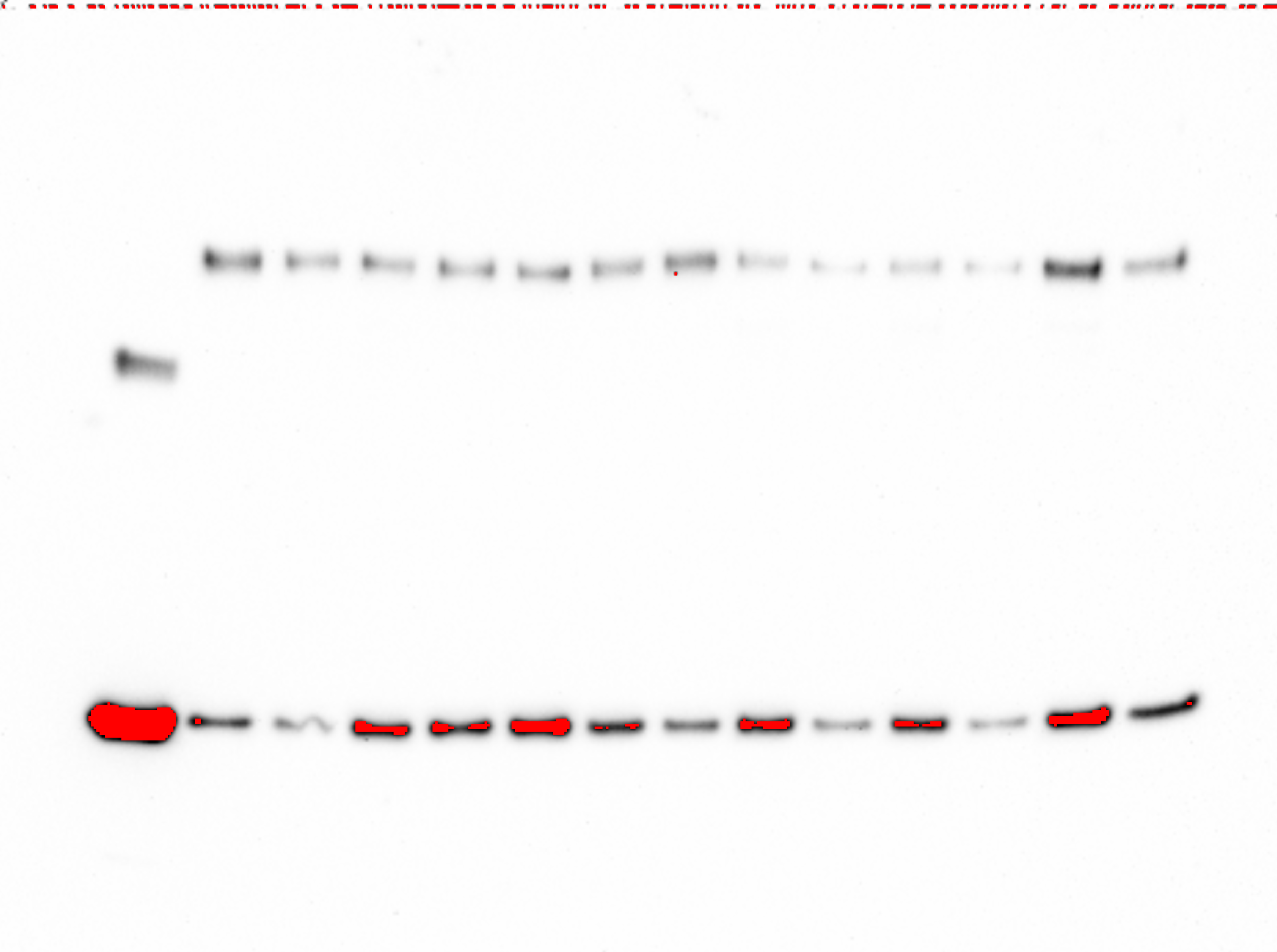

Supplement: Figure 2—figure supplement 2—source data 1. [file elife-91422-fig2-figsupp2-data1.zip › Figure2-S2B_SourceData1/Figure2-S2B_NaK_pico_300s.tif]

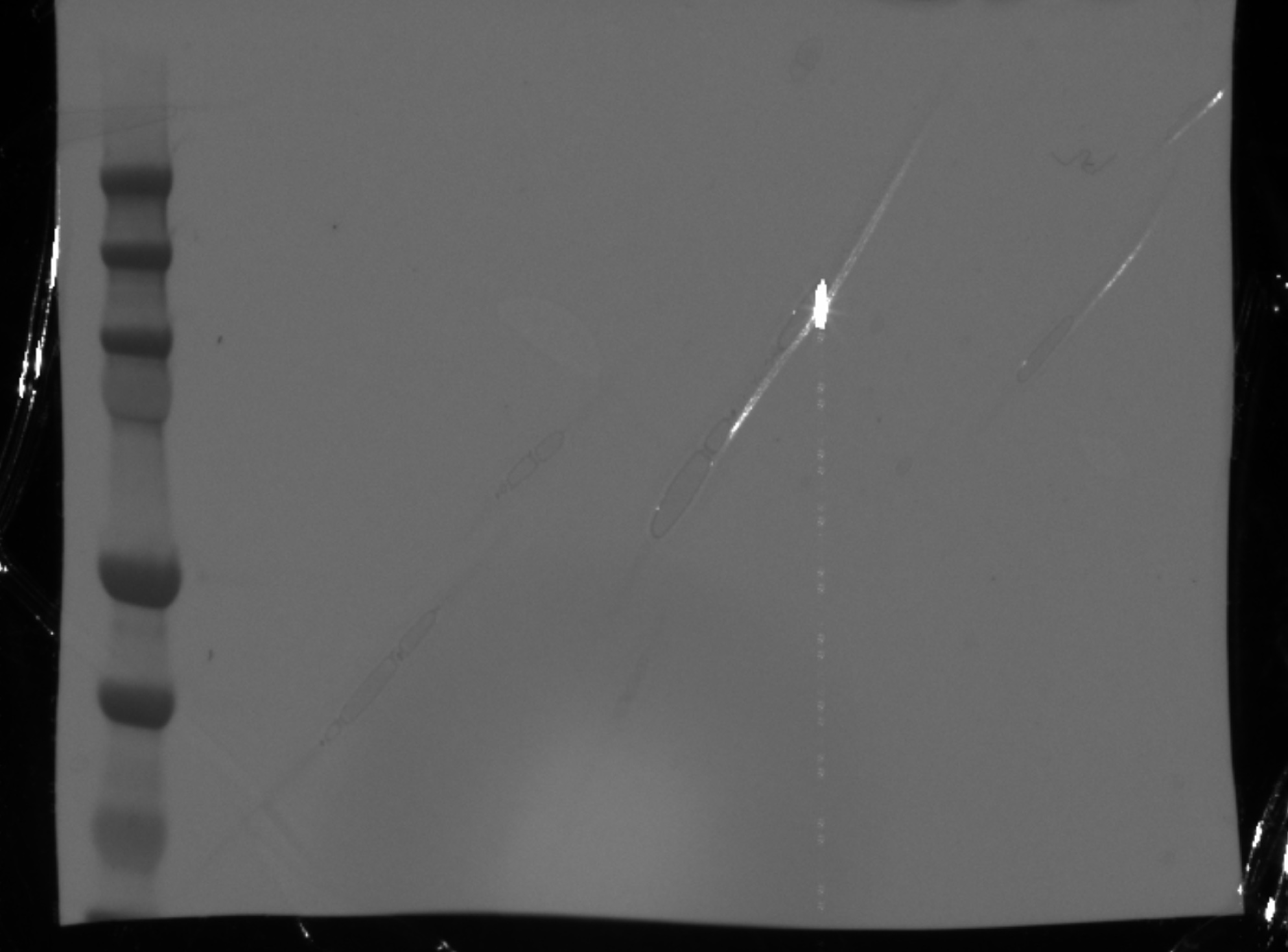

Supplement: Figure 7—figure supplement 1—source data 1. [file elife-91422-fig7-figsupp1-data1.zip › Figure7-S1_SourceData1/Figure7-S1B-left_LossOfN2Protein_ladder.jpeg]

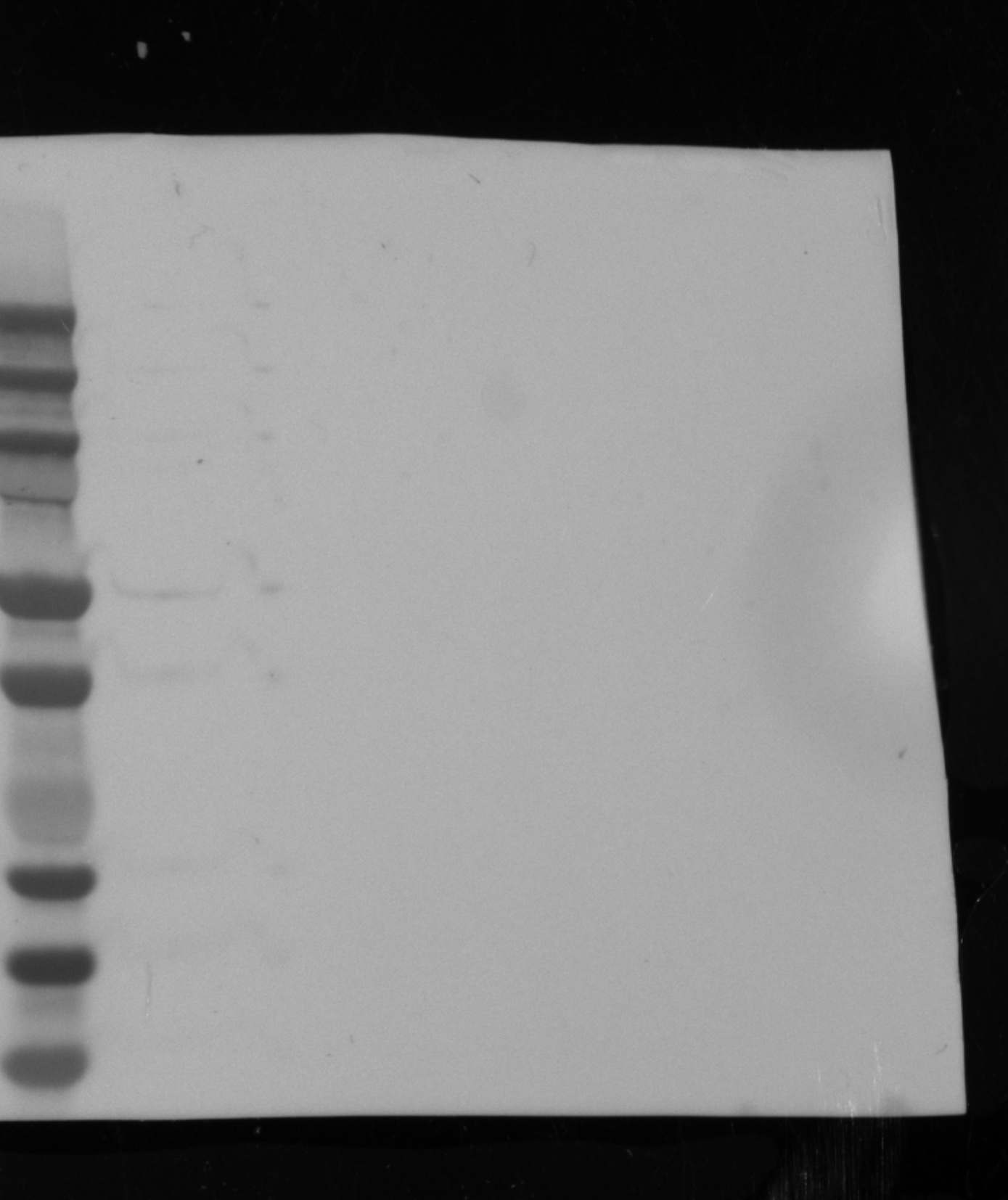

Supplement: Figure 7—figure supplement 1—source data 1. [file elife-91422-fig7-figsupp1-data1.zip › Figure7-S1_SourceData1/Figure7-S1B-right_LossOfN2Protein_ladder.jpeg]

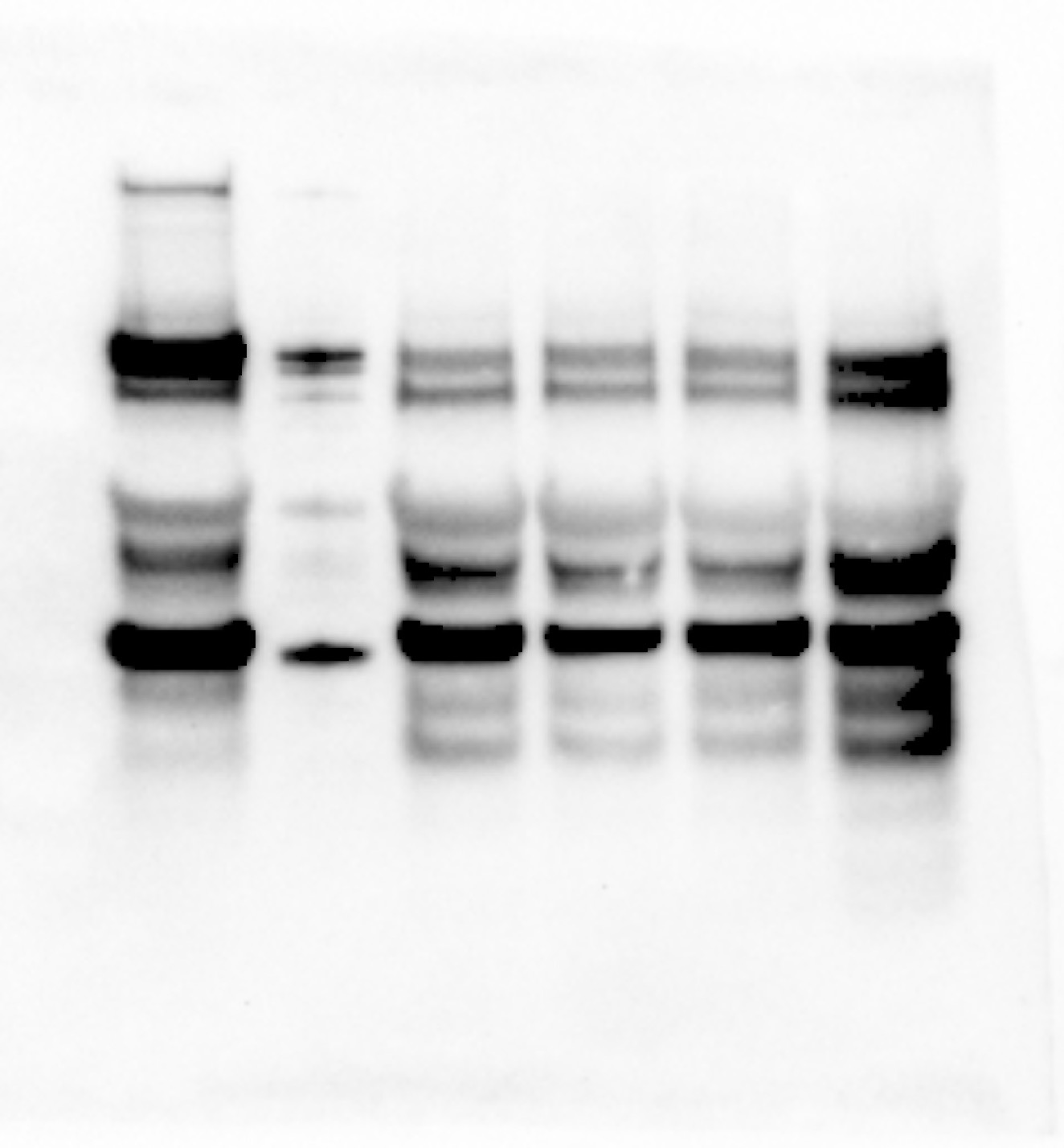

Supplement: Figure 7—figure supplement 1—source data 1. [file elife-91422-fig7-figsupp1-data1.zip › Figure7-S1_SourceData1/Figure7-S1B-right_LossOfN2Protein.jpeg]

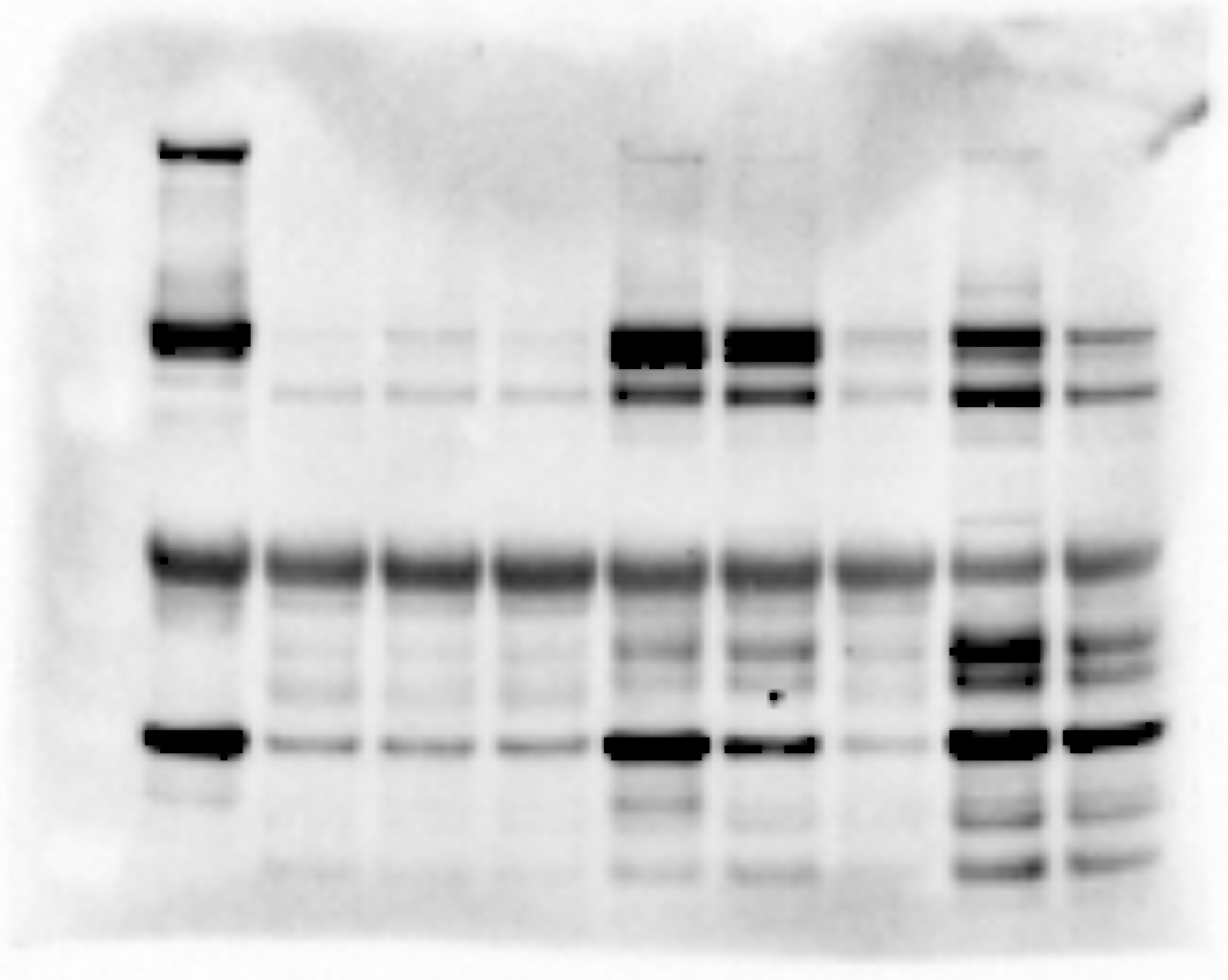

Supplement: Figure 7—figure supplement 1—source data 1. [file elife-91422-fig7-figsupp1-data1.zip › Figure7-S1_SourceData1/Figure7-S1B-left_LossOfN2Protein.jpeg]

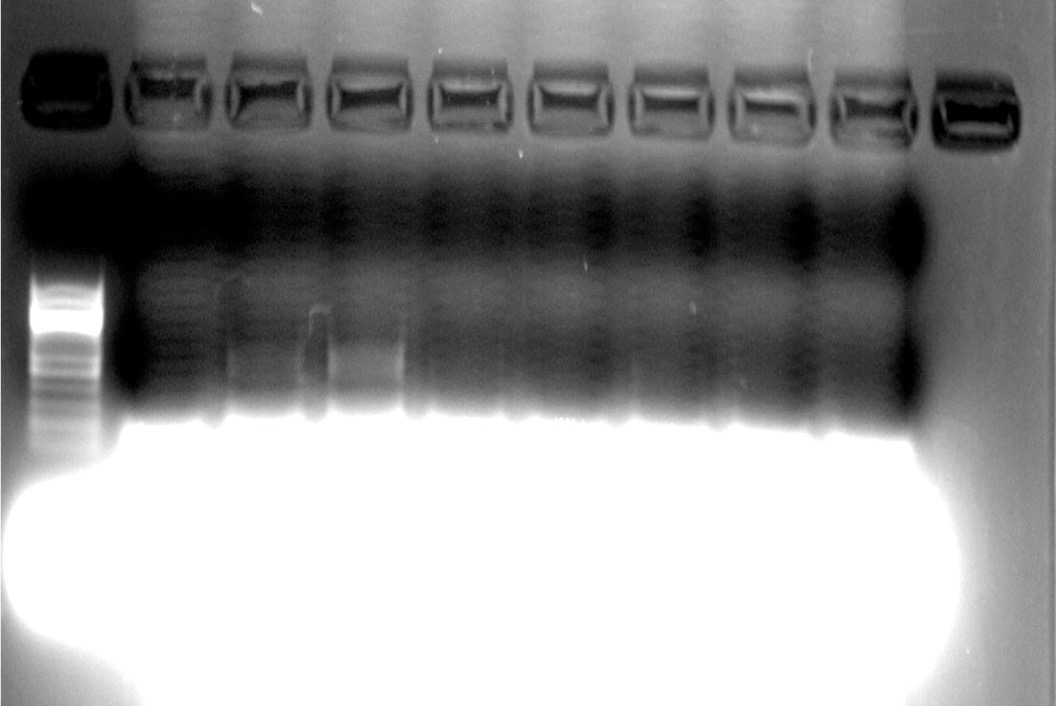

Supplement: Figure 7—figure supplement 1—source data 3. [file elife-91422-fig7-figsupp1-data3.zip › Figure7-S1C_SourceData1/Figure7-S1C_LossOfJag1Transcript.jpg]
