# Supplementary material for: Diversity in Notch ligand-receptor signaling interactions
Source: eLife. 2025 Jan 3;12:RP91422. doi: 10.7554/eLife.91422 (PMC11698495; doi:10.7554/eLife.91422)
Supplement: Figure 2—figure supplement 2—source data 2. [file elife-91422-fig2-figsupp2-data2.zip › Figure 2-figure supplement 2 -source data 2.pdf]

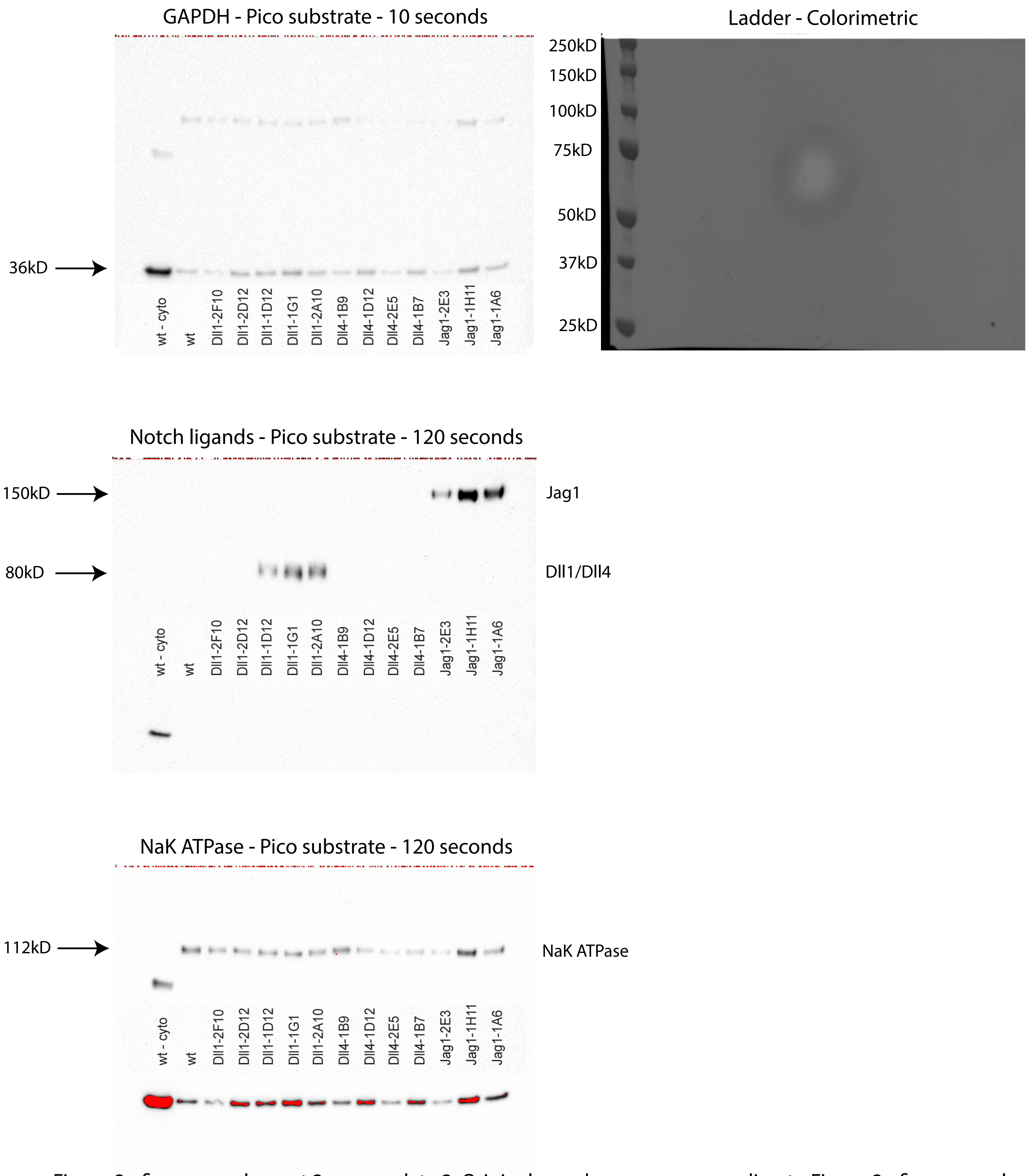

Figure 2 - figure supplement 2, source data 2. Original membranes corresponding to Figure 2 - figure supplement 2, panel B. Shows the quantification of surface ligand (Jag1, Dll1/Dll4) in CHO-K1 sender clones. GAPDH and NaK ATPase were measured as cytoplasmic fraction and surface fraction marker proteins, respectively. Precision Plus Protein Dual Color Standards (BioRad) were used for all images (upper, right blot image) .
