# Supplementary material for: Diversity in Notch ligand-receptor signaling interactions
Source: eLife. 2025 Jan 3;12:RP91422. doi: 10.7554/eLife.91422 (PMC11698495; doi:10.7554/eLife.91422)
Supplement: Figure 7—figure supplement 1—source data 2. [file elife-91422-fig7-figsupp1-data2.zip › Figure 7-figure supplement 1B -source data 2.pdf]

Notch2 and GAPDH protein detection

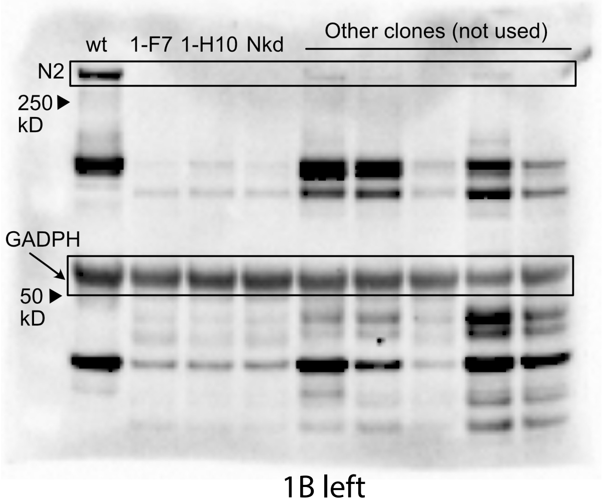

1B left

Ladder - Colorimetric

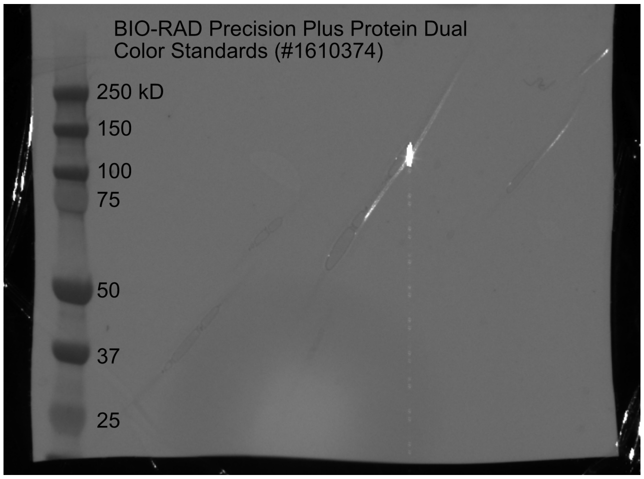

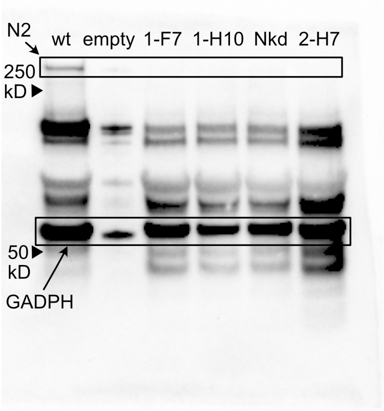

1B right

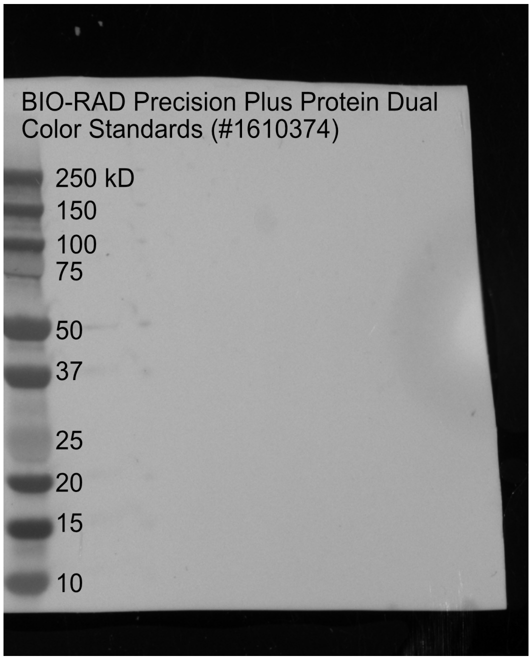

Figure 7 - figure supplement 1, source data 2. Original membranes corresponding to Figure 7 - figure supplement 1, panel B. Shows the detection or absence of endogenous Notch2 expression in CRISPR-treated C2C12 cell lines. (Top, 1B left) First Western blot performed. (Bottom, 1B right) Repeat Western blot to confirm the original results. Precision Plus Protein Dual Color Standards (BioRad) were used.
