# Supplementary material for: Diversity in Notch ligand-receptor signaling interactions
Source: eLife. 2025 Jan 3;12:RP91422. doi: 10.7554/eLife.91422 (PMC11698495; doi:10.7554/eLife.91422)
Supplement: Figure 7—figure supplement 1—source data 4. [file elife-91422-fig7-figsupp1-data4.zip › Figure7-S1C_LossOfJag1Transcript_labeled.pdf]

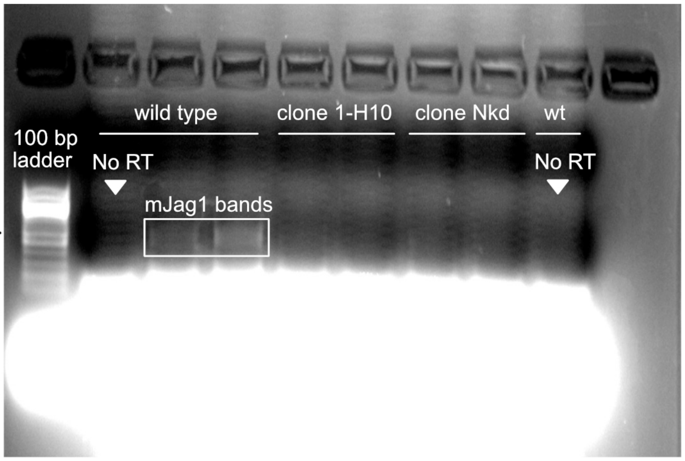

Figure 7 - figure supplement 1, source data 4. Original RT-PCR gel corresponding to Figure 7 - figure supplement 1, panel C. Shows the presence of endogenous Jag1 transcript in wildtype C2C12 cells and the absence of endogenous Jag1 transcript in CRISPR-treated C2C12 cell lines.
